# The complexity of cardiovascular long COVID: where we are

**DOI:** 10.1093/cvr/cvae090

**Published:** 2024-05-17

**Authors:** Rahul S Gandhi, Betty Raman

**Affiliations:** Wellington Cardiovascular Research Group, Wellington Hospital, Wellington, New Zealand; Radcliffe Department of Medicine, University of Oxford and Oxford University Hospital Foundation NHS Trusts, John Radcliffe Hospital, Headley Way, Oxfordshire OX3 9DU

The COVID-19 virus has left in its wake, a pandemic that has claimed over 7 million lives^1^ and caused significant injury to countless others. Whilst the advent and dissemination of COVID-19 vaccines have been critical to reducing this burden, residual pathologies remain manifest in various organ systems. Cardiovascular outcomes in COVID-19 remain a challenging topic given the high burden of cardiac symptoms, which are not always associated with objective abnormalities complicated by the lack of adequately matched control populations in studies and paucity of data for appropriate therapeutic options.

The initial demonstration of COVID-19’s harmful effects on the heart was observed through clinical reports of acute cardiac injury, which were subsequently corroborated by findings from autopsies and cardiac biopsies.^2^ Research using inducible pluripotent stem cell-derived cardiomyocyte models, cell cultures, and mouse models has been crucial in understanding COVID-19’s impact on the heart, revealing direct viral damage to cardiomyocytes.^3^ The complexity of the virus’s impact was quickly recognized during the acute phase of COVID-19 as various cardiac symptoms emerged (*Figure 1*). Patients not only exhibited myocarditis and acute coronary syndromes, indicative of endothelial involvement and vascular inflammation,^4^ but also experienced a wide array of symptoms such as arrhythmias, shortness of breath, fatigue, and reduced exercise capacity.^5^ Autonomic disturbances associated with COVID-19 were also identified, including conditions like postural orthostatic tachycardia syndrome and orthostatic hypotension. For some patients, these symptoms improved with time, yet for others, the diverse and unpredictable array of effects persisted beyond the acute phase. This led to the patient-coined term ‘Long COVID’, defined formally as the continuation of symptoms for at least 2 months after an initial SARS-CoV-2 infection and lasting beyond 3 months^6^ in the absence of other diagnoses. It is estimated that approximately 65 million individuals globally may be afflicted with post-acute sequelae of SARS-CoV-2 infection, which adversely affects their quality of life, limits function, and delays their return to work.^7^

**Figure 1:**
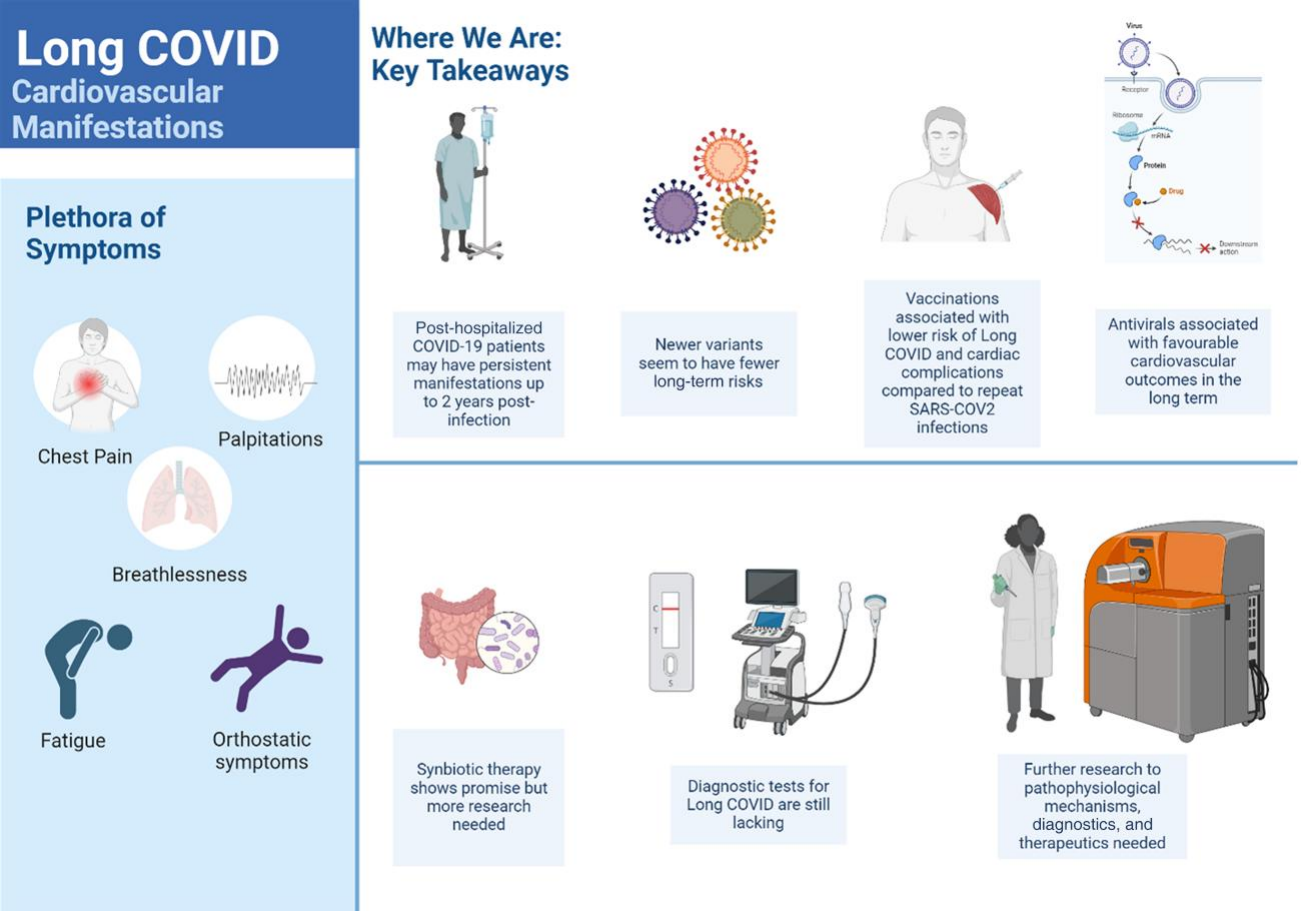
Long COVID and cardiovascular outcomes—where we are now. Central illustration demonstrates the potential for patients to continue to experience prolonged symptoms even with newer variants of SARS-CoV-2, although some improvement is seen in individuals who are vaccinated or in those who received antivirals. At present, therapeutic strategies are few, and diagnostic tests are still lacking, highlighting the need for further research into this difficult condition (created using Biorender.com).

In 2022, a study by Al-Aly *et al*.,^8^ using the Veterans Affairs Healthcare database, revealed a significant cardiovascular impact of COVID-19 in 2020–21 compared with historical controls. The research showed higher incidences of conditions like stroke, arrhythmias, myopericarditis, ischaemic heart disease, heart failure, and venous thrombosis among hospitalized (especially in unvaccinated individuals needing intensive care) and non-hospitalized COVID-19 patients. The hazard ratio for major cardiac events was 1.55 relative to non-COVID controls. These findings corroborated with a subsequent report from the UK Biobank where the increased risk of cardiovascular outcomes was particularly notable in patients previously hospitalized with COVID-19.^9^ Further analysis by the same investigators indicated that hospitalized COVID-19 patients, after 2 years from infection, continued to exhibit a heightened risk of cardiovascular complications (hazard ratios ranging from 1.45 for heart failure to 2.22 for ventricular arrhythmia), whilst this elevated risk was no longer significant in non-hospitalized post-COVID-19 patients.

During the COVID-19 pandemic, vaccine-associated complications like myocarditis and pericarditis emerged, leading to further scrutiny of the vaccination risk-benefit ratios. Supporting evidence suggested that COVID-19 mRNA vaccines might induce myocarditis through various mechanisms, including persistent mRNA, dose-dependent toxicity, spike protein effects, autoantibody-mediated damage, innate immune system activation, and genetic susceptibilities.^10^ Despite these concerns, the American College of Cardiology and European Society Of Cardiology have continued to endorse COVID-19 vaccinations, as accumulating evidence indicated that vaccine-related complications are minimal and the benefits of vaccination in preventing morbidity and mortality significantly outweigh the risks (*Figure 1*).

As the Alpha strain gave way to newer variants like Delta and Omicron, their long-term impact on cardiovascular health gained significance. A recent review hinted that despite Omicron’s higher transmissibility, myocardial infarction was less common in Omicron-infected patients, both in vaccinated and unvaccinated groups, compared with those infected with Alpha and Delta strains.^11^ Whilst Delta variants exhibited greater virulence and mortality, Antonelli *et al*.^12^ also reported a significantly lower prevalence of Long COVID in Omicron cases (odds ratio 0.3–0.5) within vaccinated cohorts, accounting for factors like age and vaccination timing. However, related data on cardiac complications specifically is still unavailable.^10^ In the broader context, vaccination appears to provide protection against Long COVID, although study outcomes vary depending on study type, vaccine timing, and virus strain. The largest report from the UK Office of National Statistics shows a 50% drop in development of Long COVID with doubly vaccinated individuals and with Omicron than with Delta strains, for instance.^13^ Importantly, these findings lend support to the hypothesis that persistent viral burden and accompanying immune response may be a trigger for Long COVID. These are further backed by observations that antiviral medications could potentially reduce the risk of severe infections and Long COVID. Specifically, nirmatrelvir, especially when combined with ritonavir, was found to decrease the prevalence of Long COVID by 26%, thereby reducing the risk of arrhythmias and ischaemic heart disease.^14^ Likewise, oral agents such as molnupiravir have been linked to a modest but significant reduction in Long COVID and arrhythmias.^15^ Recent studies have also observed a decreased incidence of Long COVID with repeated infections. However, it is worth noting that recurrent infections may still be worse than having no infection or just one prior infection with regard to Long COVID risk.^8^

At present, robust multicentre evidence for Long COVID treatments remains limited, although certain interventions offer hopeful signs. A blinded single-centre randomized controlled trial (RCT) on the synbiotic formulation (combination of prebiotic and probiotic) SIM01 revealed substantial improvements in fatigue by two- to three-fold yet only showed modest improvement in shortness of breath and no significant change in chest pain compared to placebo.^16^ A targeted metabolic modulator, AXA1125, designed to address mitochondrial dysfunction, has demonstrated potential in reducing fatigue in a preliminary prospective single-centre study.^16^ Another multicentre RCT found that metformin administered early in the infection reduced Long COVID risk by 41%.^17^ Alternative methods, such as plasma apheresis, cold water immersion therapy, and hyperbaric oxygen therapy, have also yielded varied outcomes in open-label observational studies.^5^ Patients with autonomic dysfunction appear to benefit from specific exercises, including recumbent cycling and brisk walking.^18^ Pharmacotherapies involving beta-blockers, pyridostigmine, fludrocortisone, and midodrine have been administered with inconsistent but occasionally positive effects on symptoms.^5^ Further investigations in large RCTs^19,20^ with a focus on cardiovascular outcomes are still awaited, with numerous national efforts to evaluate the impact of immunomodulatory treatments and anticoagulants still ongoing.

In summary, the complexity of Long COVID symptoms continues to pose ongoing challenges, with particular concern surrounding persistent cardiovascular complications, especially in patients who have been previously hospitalized. The full extent of the disease’s burden, including its quality-adjusted life impact, remains elusive. As millions worldwide continue to suffer, with numbers potentially rising due to new variants, the public health and economic implications are significant. The condition’s exact mechanisms are still not understood, and with a portion of the global population remaining unvaccinated, the urgency for solutions and better data remains high.

## Authors

**Biography:** Rahul S. Gandhi, BSc, MBChB, MBA, MPH, DTM&H, is interested in solving wicked global health issues in cardiology, including the long-term effects of systemic inequities and the sequelae of disparities. As an advanced trainee in cardiology at the Wellington Heart and Lung Unit under the auspices of the Royal Australasian College of Physicians, he is undertaking projects that examine the effect of indentured labour in the ancestors of Fijian Indians that is manifested as high rates of early cardiovascular disease seen throughout the South Pacific. He is a member of the research group at GANNA (Genetics of Cardio-metabolic Disease in Fijian Indians living in Aotearoa, New Zealand). After medical school in Auckland, New Zealand, he read for a Masters of Public Health at Harvard as a Fulbright Scholar, and a Masters of Business Administration at Oxford's Said Business School, and DTM&H from LSHTM. He grew up in India and Oman but has had the privilege of living in six other countries. He is deeply influenced by the life and legacy of his mentor, Professor Paul Farmer, and wishes to collaborate with like-minded cardiology colleagues in his effort to become an impactful academic cardiologist.

**Biography:** Professor Betty Raman, DPhil MBBS FRACP FESC, a recipient of the prestigious Wellcome Career Development Award, is an Associate Professor of Cardiovascular Medicine at the Radcliffe Department of Medicine, University of Oxford. She is at the forefront of innovative research into genetic heart diseases and the long-term impacts of the COVID-19 pandemic. Her leadership was instrumental during the COVID-19 pandemic, spearheading pivotal studies aimed at unravelling the enduring consequences of COVID-19 on cardiac and multi-organ health. Notably, she led one of the largest UK-wide multi-organ MRI follow-up studies (C-MORE study) examining the impact of COVID-19 on cardiac and multisystem health in post-hospitalized patients. As a highly cited figure in her field, Professor Raman continues to contribute as a co-investigator to two UK NIHR-funded national studies on Long COVID, seeking to examine the long-term impact of COVID-19 in non-hospitalized patients. She also plays a vital role as a board member in the PHOSP-COVID study, a comprehensive UK follow-up study tracking the health outcomes of 8000 patients after hospitalization due to COVID-19. In addition to these national efforts, Dr Raman also undertook one of the first clinical trials evaluating the efficacy of metabolic modulators in the treatment of Long COVID and demonstrated its potential for improving fatigue symptoms. Dr Raman’s exceptional contributions to cardiology and Long COVID research have earned her a series of national and international awards, including awards from the European Society of Cardiology and the American Heart Association. Throughout the pandemic, she has shared her profound insights and findings on Long COVID, thereby influencing the global discourse on this major public health issue.
